# Targeted salvage lymphadenectomy in patients treated with radical prostatectomy with biochemical recurrence: complete biochemical response without adjuvant therapy in patients with low volume lymph node recurrence over a long-term follow-up

**DOI:** 10.1186/s12894-015-0004-y

**Published:** 2015-02-21

**Authors:** Alexander Winter, Rolf-Peter Henke, Friedhelm Wawroschek

**Affiliations:** University Hospital for Urology, Klinikum Oldenburg, School of Medicine and Health Sciences, Carl von Ossietzky University Oldenburg, Rahel-Straus-Straße 10, 26133 Oldenburg, Germany; Oldenburg Institute of Pathology, Oldenburg, Germany

**Keywords:** Prostate cancer, Choline positron emission tomography/computed tomography, Biochemical recurrence, Salvage lymphadenectomy, Lymph node metastases

## Abstract

**Background:**

Choline positron emission tomography/computed tomography (PET/CT) represents an option in restaging of prostate cancer patients with disease relapse after local treatment. The present study assess whether salvage resection of lymph node metastases detected on choline PET/CT imaging in prostate cancer patients with biochemical recurrence after radical prostatectomy can result in a long-term complete biochemical remission, without adjuvant therapy.

**Methods:**

We analysed 13 patients with prostate specific antigen (PSA) recurrence (PSA median 1.64 ng/ml, range 0.5-9.55) after radical prostatectomy and suspicious lymph nodes (median 1; range 1–3) detected on [11C]choline and [18F]fluoroethylcholine PET/CT scans. An open salvage lymphadenectomy of positive lymph nodes in a PET/CT scan and nearby lymph nodes was carried out. We examined PSA outcome without adjuvant therapy; defined complete biochemical remission as PSA <0.01 ng/ml. Histological and PET/CT findings were compared.

**Results:**

Ten of 11 patients with histologically confirmed lymph node metastases showed a PSA response. Three of ten patients with single lymph node metastases had a complete biochemical remission (median follow-up 72 months, range 31.0-83). In five cases with single lymph node metastasis PSA decreased <0.02 ng/ml. Histologically confirmed 13 of 16 metastasis suspicious lymph nodes. No lymph node metastases were detected in two patients. All of the additionally removed 30 lymph nodes were correctly negative.

**Conclusions:**

This is the first confirmation of a complete biochemical remission after PET/CT guided secondary resection of a single lymph node metastasis in prostate cancer patients with biochemical recurrence after radical prostatectomy, over the long-term (>6.5 years), without adjuvant therapy. In order to improve these promising results, longer-term studies with more patients are required.

## Background

Biochemical recurrence following surgical treatment of prostate cancer is a common event. The introduction of new functional imaging developments have improved the detection of the site of small volume lymph node (LN) recurrence in prostate cancer patients. This raises now the question if there is a role for secondary targeted surgery of small volume LN recurrences. In recent years, different research groups have published data on positron emission tomography/computed tomography (PET/CT) guided salvage LN dissection in patients with biochemical recurrence and nodal recurrence after a radical prostatectomy [1-3]. Based on this data situation, salvage lymphadenectomy is seen as a possible option for patients with disease relapse limited to the LNs after radical prostatectomy [4]. Integrated [11C]choline or [18F]fluoroethylcholine PET/CT scans, despite their limitations [5], have exhibited good sensitivity and specificity for detecting small (>5 mm) LN metastases after radical prostatectomy. In patients with biochemical failure after local treatment with curative intent, actual meta-analysis results showed pooled sensitivity, specificity, and a diagnostic odds ratio of 85%, 88%, and 41.4 respectively, on a per-patient basis [6]. In contrast, CT and conventional magnetic resonance imaging (MRI) are not definitive for early detection of LNs recurrence. Lymphotropic nanoparticle enhanced MRI can detect smaller LN metastases (>2 mm) but has not been approved for routine diagnostics [7].

Long-term follow-up studies have shown relapse-free survival after radical prostatectomy in patients with minimal lymphatic dissemination, even without adjuvant therapy [8,9]. These data suggest that resection of LN metastases in selected patients in a secondary situation is also beneficial. However, most prior studies dealing with salvage LN dissection provide no evidence on long-term complete PSA response without adjuvant therapy. These patients were either treated after the secondary resection of LN metastases with hormones or radiation therapy [1,10] or were monitored without adjuvant therapy, mostly over a short period [11]. Moreover, these studies and Rigatti et al. [2] defined the biochemical response to treatment only as prostate specific antigen (PSA) <0.2 ng/ml.

Our group has already published promising initial results of patients with relapse-free survival after secondary LN surgery without adjuvant therapy [3]. The aim of the present study with more patients and a longer follow-up is to evaluate whether solely secondary resection of LN metastases in patients with biochemical recurrence or PSA persistence after radical prostatectomy can result in a complete long-term biochemical remission (PSA <0.01 ng/ml). The PSA outcome after targeted resection of LN metastases detected on choline PET/CT scans was analysed over the long-term in patients with biochemical recurrence or persistence after radical prostatectomy, without adjuvant therapy after salvage lymphadenectomy.

## Methods

### Patient population

This study included 13 patients with PSA recurrence or persistence after radical prostatectomy, performed within the previous three months to 10 years. A retropubic radical prostatectomy with pelvic lymphadenectomy was performed on 12 patients and a radical retropubic prostatectomy only on one patient. In our clinic, one patient underwent a sentinel guided LN dissection on both sides of the pelvic and an extended LN dissection on the right side, because of an advanced tumour; another one underwent only a sentinel guided LN dissection. The remaining patients had undergone conventional LN dissections carried out at other institutions and in one case at our clinic. All patients had LN pathological uptake on [11C]choline or [18F]fluoroethylcholine PET/CT scans and no signs of any local recurrence or distant metastasis. Furthermore, none of the patients showed any signs of ossary metastases in the skeletal scintigraphy.

### Integrated choline PET/CT imaging

**Figure 1:**
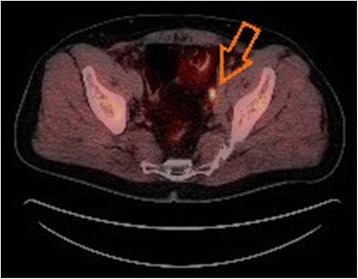
**Integrated [18F]fluoroethylcholine PET/CT scan.** Integrated [18F]fluoroethylcholine PET/CT scan shows a single lymph node metastasis in the left iliac region (arrow). The lymph node metastasis was confirmed histopathologically after secondary lymph node surgery. (Source: Clinic for Nuclear Medicine, Pius Hospital Oldenburg, Germany).

All [11C]choline or [18F]fluoroethylcholine PET/CT studies were performed in four external centres by experts using integrated PET/CT systems. Experienced radiologists and nuclear medicine specialists evaluated the images to anatomically localise the sites of pathological choline uptake. The diagnosis of tumour positive LNs on PET/CT images was based on visual evidence of the presence of focal increased uptake on a choline PET scan, where the location corresponded to LNs on CT images (Figure 1).

### Surgical procedure, PSA evaluation, histological evaluation

Two highly experienced surgeons performed open secondary LN dissections at one centre on patients in the study, between September 2004 and February 2013. They dissected pathological LNs detected on choline PET/CT scans and nearby LNs. Clavien-Dindo grading system was used to classify complications. The PSA development was monitored postoperatively without adjuvant therapy.

Androgen ablation was not continued in those patients who did have androgen ablation before the salvage surgery. Patients with PSA persistence or further PSA increase after the secondary LN surgery have been treated with hormone withdrawal in the further course and have been followed up in the study anymore. The primary histological diagnosis was made on haematoxylin and eosin-stained sections. Immunohistochemical staining of cytokeratins was performed to verify micrometastases. In one case, additional antibodies against PSA, prostate-specific acid phosphatase, p504s, and the proliferation marker Ki67 were employed for typing the metastatic tissue. The histological findings were compared with the PET/CT findings.

Since only adjacent LNs were removed in addition to the PET/CT positive LNs and furthermore, no extended lymphadenectomy was performed, it was not possible to calculate the sensitivity of the PET/CT diagnostics for detecting metastases. Specificity was calculated according to its definition (true-negatives/true-negatives + false-positives).

### Ethical approval

This study has been performed in accordance with the ethical standards and the German legislation. The current analysis represents a retrospective evaluation of a consecutive data bank, which was done by a separate unit. The data have already been made anonymous at the source. Therefore this retrospective analysis does not require formal ethical approval.

## Results

A summary of patient characteristics is shown in Table 1. In 11 of 13 patients, metastasis-suspicious LNs detected by means of PET/CT images could be completely removed. These were also histologically positive. In one patient with two metastasis-suspicious LNs detected on PET/CT scans, only one histologically-negative LN could be resected, because of severe cicatrisation. A further 30 (mean 2.3, range 0 – 10) adjacent PET/CT negative LNs were dissected and found negative for cancer. In one case, the neighbouring LNs could not be removed, because just three months earlier this patient had undergone an sentinel guided and extended pelvic lymphadenectomy on the same affected side. In another patient, only cicatricial tissue could be removed in addition to one LN metastasis after radiotherapy. In the same patient, a small lesion of the ureter necessitated secondary ureteral stenting (Clavien-Dindo IIIb). In all other cases, the intra- and postoperative courses were without complications.

**Table 1:** **Summary of patient characteristics and PSA outcome after secondary resection of lymph node metastases without adjuvant therapy**

**Patient**	**Age (yr)**	**Primary treatment**	**Initial tumour stage**	**Gleason score**	**Hormonal therapy after primary treatment**	**Radio-therapy after primary treatment**	**PSA initial (ng/ml)**	**PSA1 (ng/ml)**	**[11C] choline PET/CT**	**[18F]fluoro-ethylcholine PET/CT**	**PET/CT positive LN**	**PSA2 (ng/ml)**	**Follow-up (month)**
1	61	RRP + PLND	pT3a pN0 M0 R0	3 + 4	-	-	4.13	0.92	+	-	1	0.22	79
2	59	RRP + PLND	pT2c pN0 M0 R0	4 + 3	+	-	26.7	4.09	+	-	1	<0.01	83
3	64	RRP + PLND	pT3a pN1 M0 R0	4 + 3	-	-	16.0	2.45	+	-	1	<0.01	72
4	68	RRP	pT3a pNx M0 R0	-	-	+	9.9	1.64	-	+	1	<0.01	37
5	78	RRP + PLND	pT3b pN0 M0 R0	3 + 4	-	-	3.2	1.62	+	-	1	2.7	27
6	59	RRP + PLND	pT3a pN0 M0 R0	4 + 3	+	-	7.6	4.51	+	-	1	1.5	6
7	49	RRP + PLND	pT3b pN0 M0 R0	4 + 5	-	-	4.0	0.67	+	-	1	0.03	5
8	61	RRP + PLND	pT3a pN0 M0 R0	5 + 5	+	+	NA	9.55	+	-	3	54.46	12
9	53	RRP + PLND	pT3a pN0 M0 R1	4 + 4	-	+	36.0	3.54	-	+	1	0.4	7
10	75	RRP + PLND	pT3a pN0 M0 R1	4 + 3	-	+	5.94	3.77	+	-	1	10.3	2
11	75	RRP + PLND	pT3a pN0 M0 R0	3 + 3	-	+	7.14	0.94	-	+	1	0.01	10
12	55	RRP + PLND	pT3b pN1 M0 R0	4 + 3	-	-	5.08	0.5	+	-	2	no histologically confirmed LNM	
13	64	RRP + PLND	pT3a pN0 M0 R1	-	-	+	8.21	1.23	+	-	1	no histologically confirmed LNM	
**Median**	**61**							**1.64**			**1**		

RRP =  radical retropubic prostatectomy; PLND = pelvic lymph node dissection; PSA initial = PSA at primary diagnosis; PSA1 = PSA at time of PET/CT diagnosis; PSA2 = PSA after resection of lymph node metastases; LNM = lymph node metastases. NA = not available.

After the secondary LNs resection, ten of 11 patients with histologically-confirmed LN metastases showed a PSA response, without adjuvant therapy after secondary LN surgery. In five of ten patients with single LN metastases the PSA value decreased <0.2 ng/ml. Three patients with single metastases had a lasting complete PSA remission (<0.01 ng/ml). The maximum follow-up duration for these patients was 83 months (median 72 month, range 31.0 – 83). In one of the four cases with a single LN metastases and only a partial, incomplete remission local recurrence was detected in the course of the study by means of PET/CT and MRI. In the three other patients with incomplete remission, a tumorous infiltration of the adjacent tissue had already been detected histologically. In the patient without PSA response, three LN metastases were histologically confirmed. Table 1 shows the PSA outcome for all patients.

## Discussion

The introduction of new functional imaging techniques improved detection of LN metastases in prostate cancer and opens new focal treatment options. For example studies with magnetic resonance lymphography guided intensity-modulated radiotherapy have shown to eliminate metastatic lymph nodes [12]. The role of secondary LN surgery in prostate cancer is still being debated. In recent years, an increasing number of publications have released data on secondary LN dissections in patients treated with radical prostatectomy and radiation therapy. However, the studies did not analyse the course of PSA or a complete remission after secondary resection of LNs metastases for longer than five years, without adjuvant therapy. Moreover, these studies defined a relapse-free condition as a PSA of only 0.2 ng/ml (Table 2). Ours is the first study that has been able to confirm complete PSA remission (<0.01 ng/ml) lasting over almost seven years, following secondary LN surgery of a patient with biochemical recurrence after radical prostatectomy, without adjuvant therapy. The median follow-up period was 72 months for three relapse-free patients, all of whom exhibited minimal lymphatic dissemination or a single LN metastasis.

**Table 2:** **Biochemical response in prostate cancer patients after PET/CT guided salvage lymph node surgery**

**Literature**	**Year**	**Patients or LN dissections**	**Patients or LN dissections with positive histology**	**Positive nodes (mean)**	**PSA at time of PET/CT diagnosis (mean, ng/ml)**	**Biochemical response (%)**	**Complete biochemical response without adjuvant therapy in patients with histologically confirmed LN metastases**			
							**PSA <0.2 ng/ml**	**follow-up (month, mean)**	**PSA <0.01 ng/ml**	**follow-up (month, mean)**
Scattoni et al. [1]	2007	25	19 (76%)	8.8	1.98	NA	NA	NA	NA	NA
Rigatti et al. [2]	2011	72	60 (83%)	9.8	3.7	57	4 of 28 patients	38.9	NA	NA
Jilg et al. [10]	2012	52^†^	47 (90%)^†^	9.7	3.9	46	NA	NA	NA	NA
Martini et al. [13]	2012	8	6 (75%)	1.0	1.6	67	NA	NA	NA	NA
Winter et al.		13	11 (85%)	1.0	2.7	90	5 of 10 patients	41.4	3 of 10 patients	64 (max. 83)

^†^LN dissections.

LN = lymph node, NA = not available.

The patients in the herein examined collective only show a comparatively low metastasis load, with an average of only one removed positive LN and an average PSA value of 2.7 ng/ml (Table 2). It is likely that the very good PSA response in our investigation also results from this. All patients with complete and thus far lasting remission were cases with only one LN metastasis. As shown previously by Rigatte et al. [2] and Jilg et al. [10], presurgical PSA levels and the number of removed positive LNs are independent predictors for clinical progression after secondary removal of the LNMs. Patients with low PSA levels (<4 ng/ml), well to moderately differentiated tumours (Gleason score ≤7); minimal or LN relapse limited to the pelvis only appear to be the best suited candidates for salvage LN dissection [4]. These factors also apply to the three patients with lasting complete PSA remission in our study.

Observations in the primary situation support a therapeutic benefit, especially for patients with minimal lymphatic dissemination, too. Several publications suggest that an extended pelvic lymphadenectomy increases the likelihood of finding positive nodes and improves biochemical relapse-free survival, particularly in patients with a maximum of two positive LNs [14-16].

One limitation of secondary LN surgery in prostate cancer patients is the constrained sensitivity of currently available imaging techniques, especially for detection of small LNs metastases. Contrary to conventional MRI and CT scans, PET ([11C]choline, [11F]choline) scans offer key benefits in detecting LN metastases, especially in case of restaging of patients with biochemical failure after local treatment with curative intent and prostate cancer foci of sizes up to 5 mm [6,17]. Nonetheless, the value of this method is limited because of the frequency of smaller metastases or micrometastases [18]. However, CT and MRI are far less reliable for detecting small metastases [19]. In future, diffusion-weighted MRI could provide additional information on tumour pathophysiology, in comparison with standardised uptake values (SUV) in choline PET/CT scans [20].

A debate is underway on whether choline PET/CT imaging offers the basis for early treatment decisions for patients with PSA failure after radical prostatectomy. Picchio et al. suggest that routine use of choline PET/CT scans cannot be recommended for PSA values *<*1 ng/ml [21]. However, patients with local recurrence after radical prostatectomy are best treated by salvage radiotherapy when the PSA serum level is *<*0.5 ng/ml. Our study detected positive findings with a very low PSA value (*≥*0.67 ng/ml). Also, Scattoni et al. [1] and others [22] have shown positive results in patients with very low PSA levels (*<*1 ng/ml). A study by Castellucci et al. detected recurrent disease in 28% of patients with PSA *<*1.5 ng/ml detected by PET/CT [23]. Distant unexpected metastases were detected by PET/CT scans in 21% of the patients, whereby unnecessary local radiotherapy could be avoided. Mamede et al. [24] evaluate the role of [11]C-choline PET/CT imaging only in patients with biochemical recurrence after radical prostatectomy, showing PSA values below 0.5 ng/ml. The choline PET/CT scan was true positive in 15 of 71 (21.1%) cases. In seven of the 71 patients (9.9%), a choline uptake was observed in pelvic LNs. Nevertheless, it is evident that the positive detection rate of choline PET/CT scans depends on the PSA level and PSA kinetics. PSA and PSA doubling time are independent predictors of positive PET/CT findings. Accordingly, Giovacinni et al. observed a significant rise in positive findings, based on the PSA level (PSA 0.2 – 1 ng/ml: 19%; 1 – 3 ng/ml: 46%; >3 ng/ml: 82%) and the PSA doubling time (>6 months: 27%; 3 – 6 months: 61%; <3 months: 81%) [25]. Besides, another principal limitation of the method is the inhibitory effect of androgen deprivation therapy on choline uptake in patients with hormone sensitive prostate cancer [26]. A [68Ga] PSMA PET/CT scan appears to offer clear advantages over a choline PET/CT scan, particularly for small LN metastases. Compared to a choline PET/CT scan, a [68Ga] PSMA PET/CT scan shows LN metastases with significantly higher contrast, especially at low PSA levels [27].

Our study shows full correlation between [11C]choline PET/CT and [18F]fluoroethylcholine PET/CT images and histological findings in ten of eleven patients with a single LN metastasis (specificity 90.9%), and a specificity of 81.3% (13/16) across all patients. As far as the method is concerned, no conclusions can be drawn on the sensitivity of choline PET/CT imaging. On the contrary, Passoni et al. showed that [11C]choline PET/CT scans had a poor positive predictive value (24%) in identifying patients with single positive LNs at salvage LN dissections [28]. These patients also underwent a pelvic or pelvic and retroperitoneal LN dissection.

One limitation of the study is that no sufficient information on PSA kinetics (e.g. PSA doubling time) after radical prostatectomy was available for the included patients. However PSA doubling time coupled with the PSA parameter is an important predictor for positive PET/CT findings in a recurrence situation [25], which could not be taken into account accordingly in this analysis.

The small and heterogeneous sample size represent the main limitation of our study. Even among patients with a single LN metastasis (n= 10), only 30% of patients had a complete permanent remission (PSA <0.01 ng/ml) with undetectable PSA. However, in five (50%) of the patients with single LNs metastasis the PSA value decreased <0.2 ng/ml. Based on this data one cannot state with certainty whether patients with biochemical recurrence and minimal lymphatic dissemination after radical prostatectomy can be cured through surgery. Nonetheless, there is much to be said for this approach, considering one patient exhibited complete biochemical recurrence after secondary resection of a single LN metastases – for a period of over almost seven years by now. Hence, at least in such cases a therapeutic effect through secondary resection of LN metastases is likely. However, the results of studies with greater patient numbers and longer follow-up remain to be seen.

## Conclusions

New functional imaging techniques have opened the door for new focal treatment options in prostate cancer recurrence. This is the first confirmation of a complete PSA remission (<0,01 ng/ml) after PET/CT guided secondary resection of single LNs metastases in patients with biochemical recurrence after radical prostatectomy, over the long-term (>6.5 years) and without adjuvant therapy after salvage lymphadenectomy. This approach at least offers a therapeutic benefit in selected cases with minimal lymphatic dissemination, possibly even a cure, through secondary dissection of LN metastases. Although choline PET/CT scans have their limitations, they are currently the most reliable and routinely available diagnostic tools for detecting LN metastases in prostate cancer with biochemical recurrence after radical prostatectomy. One can look forward to future improvements through the introduction of new tracers (e.g. 68Ga-labelled PSMA ligand), especially for detecting small LN metastases at low PSA values. In order to improve this promising results, multicenter studies are required.
